# Concurrent basivertebral nerve radiofrequency ablation and vertebral augmentation as part of a multimodal palliative plan for pathological vertebral compression fracture from spine tumor: A case series of three patients

**DOI:** 10.1016/j.inpm.2026.100830

**Published:** 2026-10-30

**Authors:** Shannon Dwyer, Allyson Kouche, Yu-Tung Chen, Jimmy Wen, Arsh Alam, Jose Silva, Foad Elahi

**Affiliations:** a California Northstate University College of Medicine, United States; b Highland Hospital, Department of Medicine, United States; c Stanford University, Departments of Orthopaedic Surgery and Neurosurgery, United States; d University of California, Davis, United States; e Sutter Roseville, Department of Emergency Medicine, United States; f California Center of Pain Medicine & Rehabilitation, United States

**Keywords:** BVNRFA, Vertebral augmentation, Tumor, Vertebral compression fracture, Vertebrogenic pain, Case report

## Abstract

**Introduction:**

Pathological vertebral compression fractures from spinal tumors are a source of severe back pain and functional disability. Vertebral augmentation (VA) has been utilized for mechanical stabilization; however, pain may persist due to nociceptive signaling from the basivertebral nerve (BVN). BVN radiofrequency ablation (BVNRFA) has shown efficacy for vertebrogenic back pain associated with Modic changes, but its efficacy for oncologic/pathologic fractures remains an area of exploration.

**Case presentation:**

We present a case series of three patients with pathological vertebral compression fractures secondary to spinal tumors who underwent concurrent BVNRFA and VA. All three patients experienced immediate and sustained pain reduction with improvements in function. No peri- or post-procedural complications were observed at one-month follow-up.

**Conclusion:**

Concurrent BVNRFA and VA is a novel and synergistic approach that may provide an additional option for select patients with pathological vertebral compression fractures from a spinal tumor within palliative and interventional pain settings. Larger studies are required to clarify ideal patient selection, duration of benefit, and safety.

## 1 Introduction

Spinal metastases are common tumors involving the spine and represent a frequent occurrence of cancer-related morbidity, with pain presenting as the hallmark symptom and a major source of functional decline [1]. Spinal metastatic disease can involve the vertebral body, pedicles, posterior elements, and epidural space, which increases pathologic vertebral compression fractures (VCF), mechanical instability, and neurologic deficits from metastatic epidural spinal cord compression (MESCC) [1].

For metastasis-induced VCF, percutaneous vertebral augmentation (VA) has been used as an adjunct to radiotherapy and systemic therapy to reduce pain and improve vertebral stability [2]. Despite these interventions, persistent pain is common and multifactorial, due to tumor-associated inflammation, vertebral endplate injury, and mechanical instability. The basivertebral nerve (BVN) provides the main sensory innervation to the vertebral body and endplates, and is responsible for pain signal transmission caused by endplate injury and intraosseous inflammation. Basivertebral nerve radiofrequency ablation (BVNRFA) is a minimally invasive procedure used to disrupt BVN-mediated nociceptive signaling and has shown significant improvements in pain reduction for patients with chronic vertebrogenic low back pain [3].

This case report demonstrates the application of BVNRFA and VA for patients with advanced malignancy and severe vertebral pain from metastatic spinal disease. The patients provided informed consent to be in this study.

## 2 Case Presentation

Each patient was reviewed in the context of their systemic disease burden, neurological status, oncology characteristics, prognosis, and treatment goals via a multidisciplinary team including the primary team, palliative, oncology, neurosurgery, and pain medicine. All three patients had a Spinal Instability Neoplastic Score (SINS) score that fell within the potentially unstable category, without indication for surgical stabilization, and management was focused on palliative pain control and quality of life. BVNRFA was utilized for mechanical pain with pathological fracture, BVNRFA for a potential vertebogenic component, and other interventions were employed to address concomitant radicular, neuropathic, or focal pain generators.

## 3 Case 1

A 74-year-old male with a history of type 2 diabetes mellitus and non-small cell lung cancer (NSCLC) managed with 15mg Morphine and 5mg Oxycodone, presented in February 2025 with severe sacral pain. During the February admission, a computed tomography (CT) scan revealed a new lytic metastasis in the T2 vertebral body with possible spinal cord involvement and an enlarging lytic mass in the upper sacrum. A magnetic resonance imaging (MRI) demonstrated an osseous lesion extending from T1-T4, consistent with metastatic involvement of the spine. The patient reported numbness in the gluteal fold and inner thigh that occasionally radiates to his legs, urinary retention, and fecal incontinence.

The patient was readmitted in November for worsening back pain. Since the previous admission in February, he had undergone five rounds of chemotherapy and radiation therapy. During this admission, 10/325mg Oxycodone/Acetaminophen was given for pain control. The following day, he reported persistent mid-to lower back and sacral pain significant enough to interfere with breathing and physical activity. A repeat MRI was consistent with previous studies, but also showed a severe pathologic VCF of L1 with mild stenosis, with a SINS score of 8 (Fig. 1). The pain physician recommended BVNRFA, radiofrequency ablation (RFA) of the intercostal nerve, and VA of the L1 vertebrae.

**Fig. 1:**
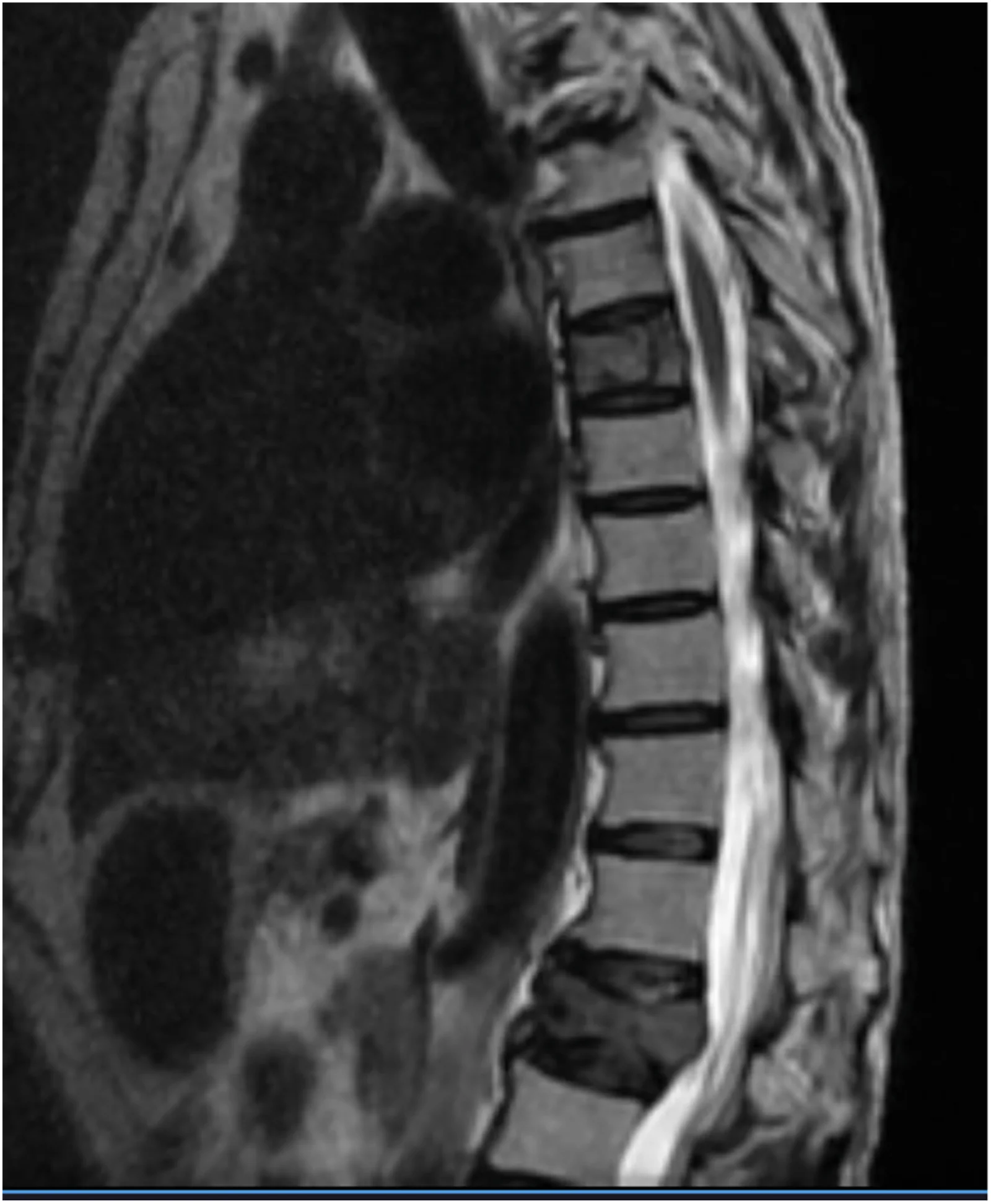
MRI showing compression fracture at T7 and L1 and osseous lesion from T1-T4.

Fluoroscopic guidance was used to identify the vertebral levels, and five needles were placed via a transpedicular approach at levels T7, L1, L3, S1, and S2. The trocar and cannula were advanced into the posterior vertebral body, followed by advancement of the hand drill to the anterior third of the body under lateral fluoroscopic guidance. The radiofrequency device was inserted, and ablation was performed for 7 minutes at 90°C. A balloon was then utilized at L1 and L3, and polymethylmethacrylate (PMMA) was injected. Following this, radiofrequency was applied at intercostal nerves VI, VII, and VIII on the left side for 90 seconds at 90°C. There were no complications during the procedure.

Post-procedure, the patient reported a pain reduction from 10/10 at baseline to 3/10. At one-month follow-up, pain was maintained at 3/10 and reduced opioid requirements.

## 4 Case 2

A 58-year-old female with a history of hypertension, anxiety, depression, and stage IV NSCLC with metastases to the spine and hip treated with chemotherapy, Morphine, and Hydrocodone/Acetaminophen, presented in November 2025 with two days of worsening right leg pain without numbness, tingling, or weakness. Computed tomography angiography (CTA) of the chest showed no pulmonary embolism, and MRI of the lumbar spine demonstrated a chronic pathologic compression deformity at L2 with retropulsion and moderate spinal canal stenosis (Fig. 2). Multiple lesions involving the lower thoracic spine, lumbar spine, and pelvic bones appear new or worsened compared with a previous positron emission tomography (PET) scan, including a sacral lesion with associated epidural disease. The patient was admitted to the emergency department for severe right leg pain and received 10mg Hydrocodone/Acetaminophen and 30 mg Morphine.

**Fig. 2:**
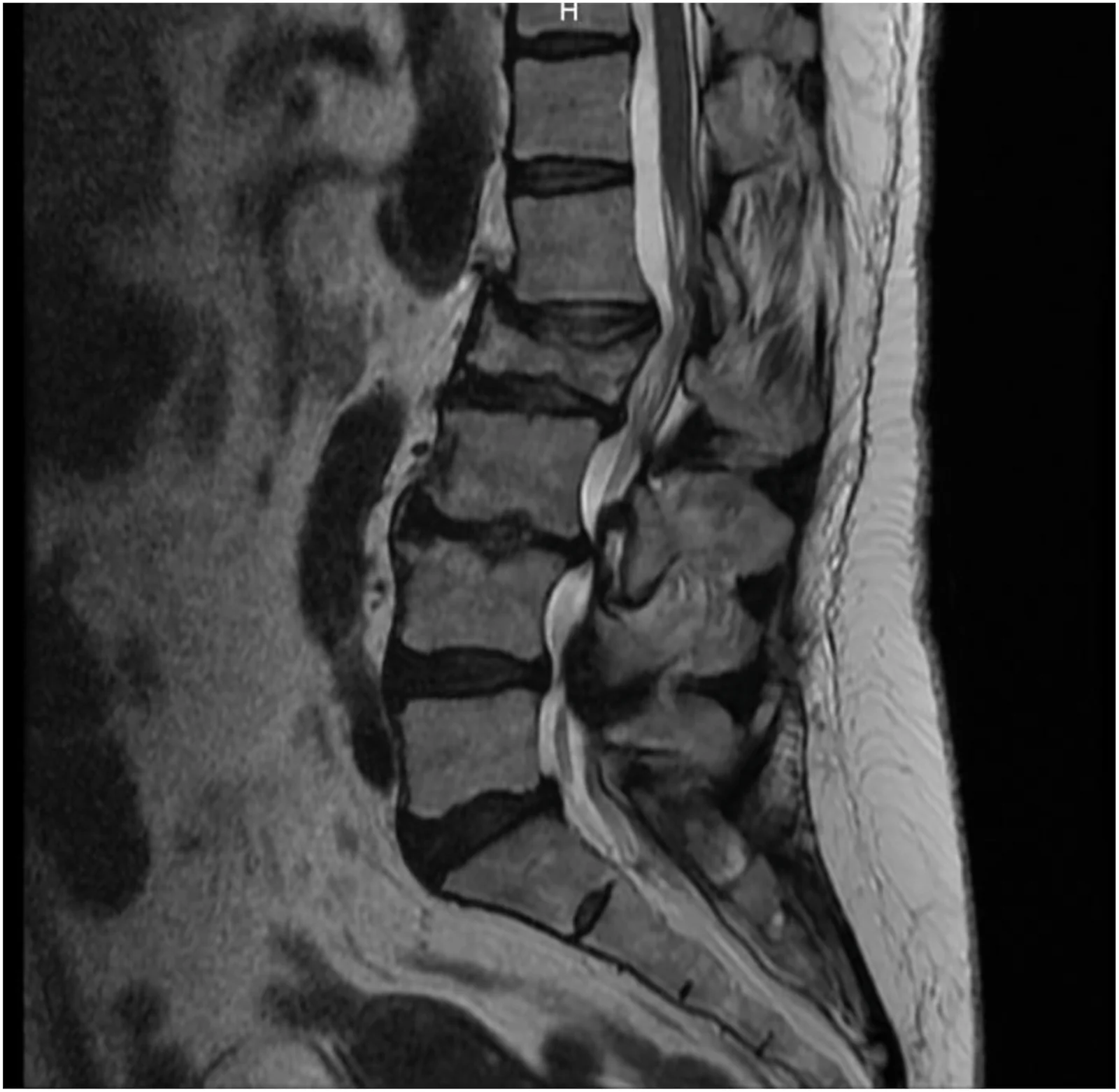
MRI showing compression fracture of L2 vertebra.

Two days later, the patient presented again with severe bilateral lower back pain without accompanying weakness. A repeat MRI was consistent with the prior study, with a SINS score of 10. The pain physician recommended BVNRFA and VA at L2, along with L4, L5, and S1 transforaminal epidural steroid injections to address the L2 pathological fracture with retropulsion and vertebral collapse.

The patient underwent BVNRFA and VA at L2. Transforaminal epidural steroid injections containing 1% lidocaine and 0.25% Marcaine were administered bilaterally at L4, L5, and S1. There were no complications, and the patient tolerated the procedure well. During a follow-up the next day, she reported significant improvement in pain and did not request any medication during the previous night.

At one-month follow-up, pain was reduced from 10/10 at baseline to 4/10. The patient reported improved ambulation and decreased opioid requirements.

## 5 Case 3

A 51-year-old male with metastatic prostate cancer to the spine presented in November 2025 with several months of back pain along with numbness and cramping in bilateral lower extremities. An MRI of the lumbar spine showed pathologic VCFs at L2 and L5, moderate spinal canal stenosis at L4-L5, and severe spinal canal stenosis and cord compression at T1 (Fig. 3).

**Fig. 3:**
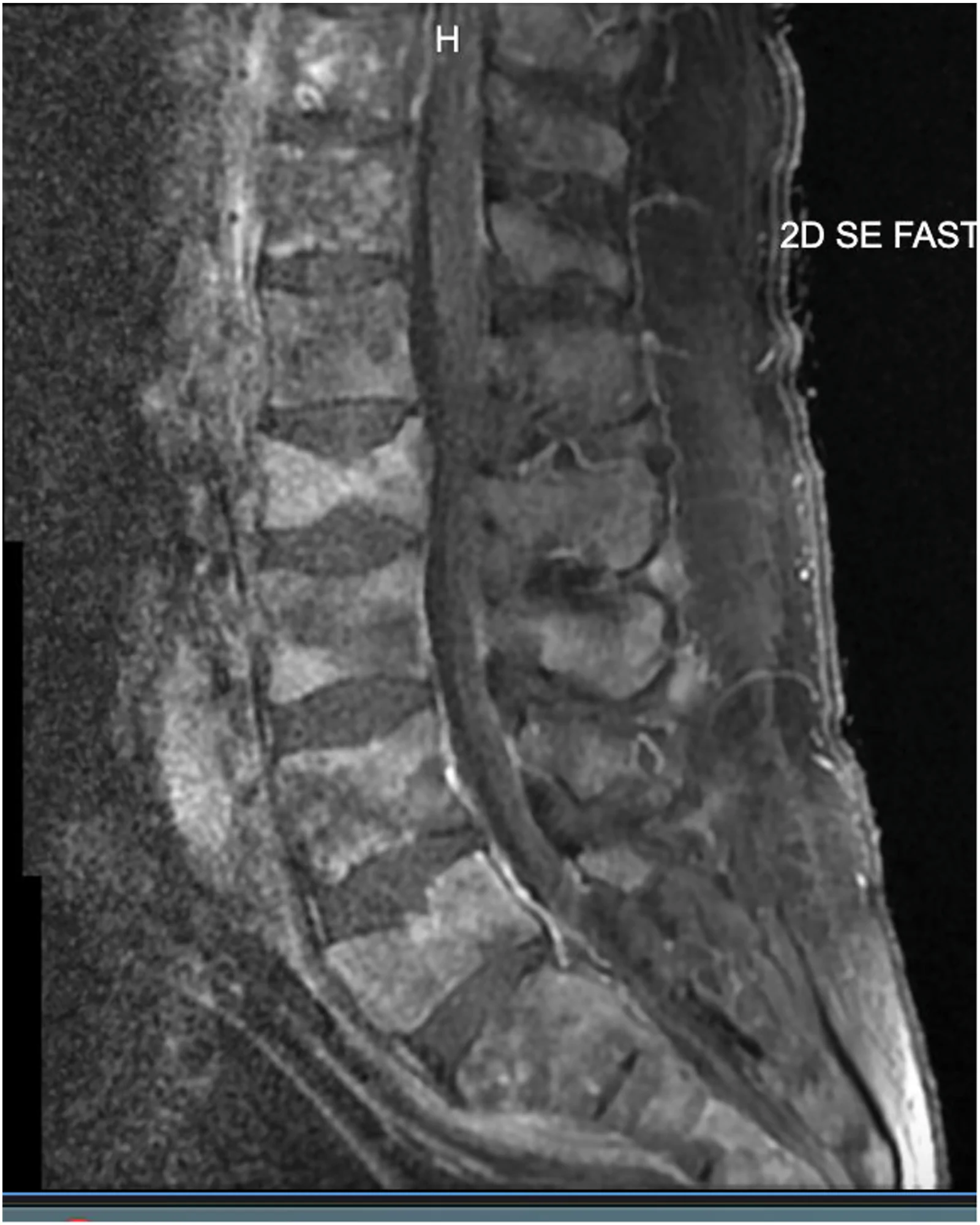
MRI showing compression fractures of L2 and L5 vertebrae.

Three days later, the patient experienced worsening weakness of the lower extremity along with loss of bowel and bladder control. An MRI demonstrated additional VCFs of the T3-L2 vertebrae, with a SINS score of 8. Based on these findings, the patient was recommended for BVNRFA and VA for T1, T5, L2, and L5, along with epidural steroid injection for T1-T2 and bilateral dorsal root ganglion radiofrequency nerve ablation for T1-T2.

BVNRFA and VA were performed at T1, T5, L2, and L5. A needle was subsequently guided fluoroscopically to T1-T2 for the interlaminar epidural steroid injection. Finally, the T1-T2 levels were again identified under fluoroscopy, and a radiofrequency needle was advanced into the foramen to target the bilateral dorsal root ganglion. 1% Lidocaine and 0.25% Marcaine were slowly injected, and ablation was performed at 80°C for 90 seconds, followed by a second ablation at 80°C for 90 seconds. All steps of the procedure were completed without complications and were well tolerated by the patient. At a follow-up the next day, the patient was able to twist and move his legs in ways he had not been able to before the procedure. He reported significant improvement in pain, with only mild residual discomfort in the upper thoracic region.

At one-month follow-up, the patient reported improved pain from a baseline of 9/10 to 3/10 with greatly reduced opioid usage.

## 6 Discussion

This case series describes the novel usage of BVNRFA and VA for treating back pain caused by pathologic VCF secondary to spinal tumors. All three patients had SINS scores reflecting a potentially unstable category ranging from 8 to 10, highlighting the complexity of decision-making and the requirement of a multidisciplinary team. BVNRFA is more commonly used to treat vertebrogenic back pain with Modic changes; however, this application was used for a pathologic VCF. VA relieves back pain by providing structural reinforcement by injecting PMMA into the fractured vertebra. A secondary mechanism may involve exothermic polymerization of PMMA, which ablates intraosseous nerve endings in the vertebral body [4]. Thus, BVNRFA with VA targets nociceptive pain signals from the BVN and mechanically stabilizes the VCF. This technique showed significant improvement in patient pain and function immediately and at one-month follow-up. VA was used to target the mechanical pain from pathologic vertebral compression and collapse, while BVNRFA was utilized to target a potential vertebrogenic source of pain. Additionally, interventions such as epidural steroid injections, intercostal nerve RFA, and dorsal root ganglion RFA were performed for clinically indicated concomitant pain generators, including radicular, thoracic, and neuropathic, respectively. The use of these additional interventions was individualized to the patient's overall clinical picture and treatment goals. Contemporary decision-making frameworks for spinal metastases, such as the NOMS (neurologic, oncologic, mechanical, and systemic) classification system, help provide a multidisciplinary guide to optimize functional outcomes, reduce morbidity, and expand palliative options to personalize care within this field [5]. Thus, for patients in this case series, treatment was selected after a goals-of-care discussion aimed at improving quality of life and palliation while avoiding more extensive or invasive interventions.

Pain caused by metastatic VCF is multifactorial. One mechanism is tumor growth within the vertebral body that increases pressure on the endosteum. This triggers an inflammatory response that activates nociceptive signals via the interosseous BVNs [6]. There is a release of inflammatory mediators (protons, bradykinin, endothelins, prostaglandins, proteases, and neurotrophins) from cancer cells and stromal cells [7]. Furthermore, osteoclastic bone resorption creates an acidic environment that causes pain by activating acid-sensing ion channels and TRPV1 receptors on the sensory neurons innervating bone [8]. Additionally, VCFs generate mechanical pain through spinal instability, which causes periosteal stretching and muscle spasm [9]. If vertebral collapse occurs, displacement of bone or tumor fragments leads to compression of the spinal cord or nerve roots [10]. For pathologic VCF from metastatic disease, VA showed clinically significant pain reduction compared to nonsurgical management or RFA alone [11]. BVNRFA can provide additional pain relief by targeting the nociceptive signals from the BVN.

Notably, there were no peri- or post-operative complications observed in this case series. Additionally, this concurrent procedure did not interfere with oncologic management. However, patient selection is critical, and factors to take into account before proceeding are spinal stability, infection risk, extent of vertebral body damage, specific tumor type, and prognosis.

This study's main limitations are the small sample size, retrospective nature, and short-term follow-up. It does not comment on the long-term efficacy of BVNRFA and VA for pain relief in pathologic VCF. Future research should involve larger patient populations, evaluate long-term pain relief, utilize standardized outcomes, and assess adverse effects in tumor-infiltrated vertebrae. Another important limitation in interpreting this case series is that the additional interventions done on top of BVNRFA and VA were performed because these patients had complex cancer-related pain with multiple potential pain generators. These interventions were performed as part of a multimodal palliative approach. Therefore, we cannot independently determine pain improvements attributable to BVNRFA and VA, and this case series was not designed to analyze the pain-reduction contribution of each intervention.

## 7 Conclusion

Concurrent BVNRFA and VA may be an efficacious and safe option for select patients with pathological VCF secondary to spinal tumors within a broader multidisciplinary palliative and interventional pain plan. This combined approach targets both mechanical and neurogenic pain, offering meaningful pain relief, especially in a palliative setting.

## Informed consent

Informed consent was obtained. All research was conducted in accordance with the Declaration of Helsinki.

No financial or material support was provided.

## Data availability statement

Data sharing is not applicable to this article as no datasets were generated or analyzed during the current study.

## Funding statement

All authors declare that no funding was received.

## Declaration of competing interest

All authors declare no competing interest.
